# Quality assurance of drugs used in clinical trials: proposal for adapting guidelines

**DOI:** 10.1136/bmj.h602

**Published:** 2015-02-25

**Authors:** Paul N Newton, David Schellenberg, Elizabeth A Ashley, Raffaella Ravinetto, Michael D Green, Feiko O ter Kuile, Patricia Tabernero, Nicholas J White, Philippe J Guerin

**Affiliations:** 1 Lao-Oxford-Mahosot Hospital-Wellcome Trust Research Unit, Microbiology Laboratory, Mahosot Hospital, Vientiane, Lao People’s Democratic Republic; 2 London School of Hygiene and Tropical Medicine, London, UK; 3 Mahidol Oxford Research Unit, Faculty of Tropical Medicine, Mahidol University, Bangkok, Thailand; 4 Clinical Sciences Department, Institute of Tropical Medicine, Antwerp, Belgium; 5 Centers for Disease Control and Prevention, Atlanta, GA, USA; 6 Liverpool School of Tropical Medicine, Pembroke Place, Liverpool, UK; 7 Worldwide Antimalarial Resistance Network, Centre for Tropical Medicine and Global Health, Oxford University, UK; 8 Centre for Tropical Medicine and Global Health, Nuffield Department of Medicine, Churchill Hospital, Oxford University, UK

## Abstract

**Paul Newton and colleagues** propose that clinical trial guidelines should include a requirement to assess and state the quality of the drugs and other medical products used

Although it is increasingly clear that substandard and falsified drugs and medical products (including devices) are an enormous public health problem,^1^ ^2^ particularly in the developing world, clinical trials have largely been considered immune from the problem.

Clinical trial guidelines from the World Health Organization and the International Conference on Harmonisation of Technical Requirements for Registration of Pharmaceuticals for Human Use (ICH) require compliance with applicable good manufacturing practices for all investigational drugs and comparators.^3^ ^4^ But they have not been updated since the 1990s so they do not include adequate consideration of the current challenges of the international drug market, where globalised production and insufficient regulatory overview have resulted in variable drug quality.^5^ ^6^

There are many different types of poor quality drugs that could mistakenly be included in clinical trials, including those with no, too little, or too much active pharmaceutical ingredient, those with the wrong active pharmaceutical ingredient, those with inadequate bioavailability, and those that degrade with toxic products or contaminants.^1^ ^2^ ^6^ ^7^ We argue that clinical trial guidelines (CONSORT, SPIRIT, STARD, and TIDieR) should include statements on the checking and reporting of the quality of drugs and medical products used in clinical research. The WHO and ICH guidelines should also be updated to include such recommendations.

## Scope of problem

We are aware of several examples of clinical trials that have been conducted or planned using drugs that did not contain what they were stated to contain. Idindili and colleagues conducted a double blind, randomised, two arm study comparing the safety and efficacy of low dose versus high dose vitamin A supplementation in young Tanzanian infants.^8^ The vitamin A capsules used were manufactured by companies in Canada and Italy. Samples were checked “regularly” during the trial for vitamin A content. Within 13 months of the trial starting the 50<thin>000 IU capsules had degraded to 32% of the expected amount of vitamin A, despite being stored under appropriate conditions. The analyses were adjusted for this major confounder, and the authors emphasised the need for quality assurance checks of such capsules in clinical trials and routine supplementation programmes.

In 2011 a study of the antimalarial drug sulfadoxine-pyrimethamine in pregnant women in six countries in Africa was planned using four locally available brands. These were tested at the US Centers for Disease Control and Prevention, and the sulfadoxine content of one of the brands was found to be below 90% of the manufacturer’s stated amount. An emergency request to procure a good quality brand was made and the study proceeded with confidence (F ter Kuile, personal communication, 2015).

Developing countries have limited regulatory capacity for verifying bioequivalence.^9^ Drugs containing the same stated active pharmaceutical ingredient are not necessarily bioequivalent if they are produced by different companies or in different batches. For example, different batches of mefloquine and digoxin tablets have shown non-bioequivalence.^10^ ^11^ ^12^ ^13^ Thus, clinical trials using different brands or batches of the same drug may give different results, which makes interpretation difficult and can dilute the power of meta-analyses that underlie many policy recommendations. Sowunmi and colleagues compared the bioavailability of three brands of oral quinine commonly available in Nigeria in a formal, randomised, crossover study in healthy adults.^14^ Alarmingly they found that one of the brands contained no detectable quinine. This product could well have been used in a clinical trial of the efficacy of quinine as an antimalarial drug in Nigeria.

The problem is not confined to developing countries. In 2007 a consignment of clopidogrel labelled as Plavix worth £1m (€1.3m; $1.5m) for use as a comparator in a clinical trial in the US was found to be falsified. After initial tests of tablet weight suggested an abnormality, formal chemical analysis demonstrated that the tablets contained only 50-80% clopidogrel. An alternative source of quality assured clopidogrel was used in the trial. The people responsible for this fraud were linked to the importation of two million doses of falsified clopidogrel, olanzapine, and bicalutamide into the United Kingdom. One of the ringleaders was sentenced to eight years’ imprisonment.^15^

If the drugs in these examples had not been tested the clinical trials may have harmed patients, concluded with erroneous results, and been a major waste of time and resources. Moreover, they may have inappropriately informed public health policy. Although there are examples of poor quality vaccines and diagnostic tests,^1^ ^2^ we were unable to find examples of their use in trials. We suspect that such trials do exist and that their inconsistent results might contribute to the large number of clinical trials not published.^16^ We argue that it is unethical to recruit patients to clinical trials without appropriate checks to ensure the adequate quality of drugs and other medical products used.

## Placebo ingredients

A related point is the inclusion of the details of placebo composition in clinical trial reports.^17^ For example, placebo contraceptive pills made of wheat flour, for use within factories, wrongly found their way into the drug distribution system in Brazil and resulted in about 200 unplanned pregnancies.^18^ If inadvertently used in clinical trials these might cause adverse events in people with coeliac disease. Similarly placebos that contain lactose may cause abdominal pain and diarrhoea in patients who are intolerant to lactose, potentially obscuring the incidence of these symptoms in the intervention arm. The use of olive oil and corn oil in placebos in trials of cholesterol lowering drugs could also skew the results. Golomb and colleagues found that only 8% of clinical trial reports published in four medical journals with high impact factors stated the placebo content and argued that it should be included in the CONSORT guidelines.^17^

## Guideline adaptation

To ensure that the drugs and medical products used in clinical trials are of good quality, we suggest that the CONSORT, SPIRIT, STARD, and TIDieR guidelines are adapted and the WHO and ICH good clinical practice guidelines are updated.^3^ ^4^ ^19^ ^20^ ^21^ ^22^

The WHO and ICH documents do mention the quality of drugs. The ICH good clinical practice guidelines include the requirements to ensure stability of investigational product, to keep sufficient quantities to reconfirm specifications if needed, and to describe excipients.^4^

The CONSORT guidelines have been widely adopted to guide the design, conduct, and reporting of clinical trials.^22^ The SPIRIT recommendations provide guidance for clinical trial protocols.^23^ The STARD initiative strives to improve reporting of studies of diagnostic accuracy, and TIDieR guidelines are aimed at the description of interventions.^24^ ^25^ They have been influential in improving practice and ensuring that trial results appropriately guide public policy. However, the quality of drugs and other medical products has not been considered.

We suggest that these guidelines include a requirement to state the quality of drugs and medical products (including diagnostic tests evaluated in trials or used for assessing inclusion criteria or endpoints). Batch numbers and expiry dates of drugs should also be stated in trial reports and the manufacturers’ certificate of analysis should be published as supplementary material.

Details of relevant approvals and authorisation numbers from stringent regulatory authorities (SRAs) should be included for drugs and placebos in published trial reports. SRAs are the few national medicine regulatory authorities that are members, observers, or associates of the ICH.^26^ If drugs used in the trial were not manufactured in a country with an SRA or without such approval the researchers should provide independent chemical analysis results (including dissolution if appropriate), and the analytical methods and results should be given in the supplementary material. Repeat assays should be performed at intervals during the trial to check for degradation.

For trials with a placebo arm the manufacturer details, including approval of the manufacturing site and ingredients from the medicines regulatory authority, should be stated. Investigators should be advised to use only one batch of each drug in clinical trials if possible. If different batch numbers are used sequentially, trial outcomes stratified by batch should be given in supplementary material. Changes between batches when used sequentially may represent a “hidden effect of time,” as would the deleterious effects of poor storage on medical products.^27^ It is not expected that the trial should be powered to allow stratified analysis per batches.

## Infrastructural changes

Although these proposals would add a further, albeit mild, additional reporting requirement, we believe that they are essential, as this critical weak link in the chain of evidence has not been addressed. Independent evaluation of trial drugs and placebos before trials commence and during trials is the only way to prevent inappropriately informed public health policy.

Systems would be required to allow trialists to find an analytical laboratory that can provide timely results. Measurement of blood or plasma drug concentrations should be encouraged. The costs of analyses will need to be added to funding applications, and grant awarding bodies should insist on the use and documentation of quality assured drugs and medical products. These proposed changes will take time and require the development of awareness and infrastructure.

Regulatory supervision as well as analytical capacity in the developing world desperately need strengthening. However, in the meantime if such testing and documentation is not possible, the lack of such evidence should be discussed in the report as a limitation of the study. If drug quality problems are discovered they should be reported to the appropriate regulatory authority and the WHO Rapid Alert System.^28^

Enormous investment in trials will be wasted and their interpretation into public policy incorrect if the quality of drugs and medical products is not assured. Effective drugs may gain a poor reputation, and the systematic reviews that dominate policy making may generate incorrect assessments of heterogeneity or even conclusions. Patients in clinical trials, like those in routine healthcare, should be offered good quality assured products.

**Key messages**

Although substandard and falsified drugs are an enormous public health problem, particularly in the developing world, their inclusion in clinical trials has been neglected
Examples from clinical research include the degradation of vitamin A capsules, poor quality sulfadoxine-pyrimethamine for a study of malaria in pregnancy in Africa, and falsified clopidogrel in the US
Clinical trial guidelines (CONSORT, SPIRIT, STARD, TIDieR) and good clinical practice guidelines from WHO and ICH should include a requirement to determine and state the quality of drugs
This will require increased awareness and development of infrastructure for accessible analytical capacity. The costs of analyses will need to be added to trial funding applications, and grant awarding bodies should insist on the use and documentation of quality assured drugs

## Notes

Contributors and sources: PNN wrote the first draft, coordinated the paper and liaised with MHRA; DS provided examples and contributed to the manuscript; EAA contributed to the manuscript; RR contributed to the manuscript; MDG contributed to the manuscript; FOtK contributed to the manuscript; PT contributed to the manuscript; NJW contributed to the manuscript, PJG had the original idea, provided examples, and contributed to the manuscript. All authors have agreed with submission and seen the final manuscript. PNN is the guarantor of the article.

Provenance and peer review: Not commissioned; externally peer reviewed.
